# *Trypanosoma brucei brucei*: Endocytic recycling is important for mouse infectivity

**DOI:** 10.1016/j.exppara.2011.01.001

**Published:** 2011-04

**Authors:** Senthil Kumar A. Natesan, Alana Black, Keith R. Matthews, Jeremy C. Mottram, Mark C. Field

**Affiliations:** a Department of Pathology, Tennis Court Road, University of Cambridge, Cambridge CB2 1QP, UK; b Institute of Infection and Immunology Research, University of Edinburgh, West Mains Road, Edinburgh EH9 3JT, UK; c Wellcome Trust Centre for Molecular Parasitology, Institute of Infection, Immunity and Inflammation, College of Medical, Veterinary and Life Sciences, University of Glasgow, 120 University Place, Glasgow G12 8TA, UK

**Keywords:** Trypanosome, Protein transport, Vesicle trafficking, Endocytosis, Recycling

## Abstract

Endocytosis in the African trypanosome, *Trypanosoma brucei*, is intimately involved in maintaining homeostasis of the cell surface proteome, morphology of the flagellar pocket and has recently been demonstrated as a bona fide drug target. RNAi-mediated knockdown of many factors required for endocytic transport, including several small GTPases, the major coat protein clathrin and a clathrin-associated receptor, epsinR, results in rapid cell death in vitro. Rapid loss of viability in vitro precludes meaningful investigation by RNAi of the roles of trypanosome endocytosis in vivo. Here we have sought to address this issue using strategies designed to produce milder effects on the endocytic system than complete functional ablation. We created a trypanosome clathrin heavy chain hemizygote and several lines expressing mutant forms of Rab5 and Rab11, described previously. All are viable in in vitro culture, with negligible impact to proliferative rates or cell cycle. Clathrin hemizygotes express clathrin heavy chain at ∼50% of wild type levels, but despite this demonstrate no defect to growth in mice, while none of the Rab5 mutants affected proliferation in vivo, despite clear evidence for effects on endocytosis. By contrast we find that expressing a dominantly active Rab11 mutant led to compromised growth in mice. These data indicate that trypanosomes likely tolerate the effects of partly decreased clathrin expression and alterations in early endocytosis, but are more sensitive to alterations in the recycling arm of the pathway.

## Graphical abstract

**Graphical abstract:**

**Figure:**
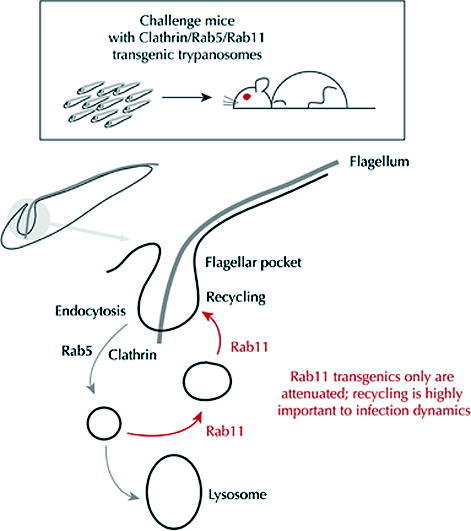

**Highlights:**

► Trypanosomes evade the immune response by antigenic variation. ► Trypanosomes also remove immune effectors from the surface by endocytosis. ► Partially defective endocytic uptake does not compromise mouse infectivity. ► Recycling pathway defects do compromise mouse infectivity.

## 1 Introduction

Maintaining the cell surface composition and turnover of surface components is a vital aspect of the cell biology of African trypanosomes. All endocytosis is mediated via uptake at the flagellar pocket, a small invagination at the posterior end of these cells and is by an exclusively clathrin-dependent, AP2-independent mechanism (Allen et al., 2003; Field and Carrington, 2009). Further, in bloodstream stages an efficient mechanism for removal of surface antibody, recognizing the dominant surface antigen, variant surface glycoprotein (VSG), also exists, and which may play a subsidiary role in immune evasion to antigenic variation (Barry, 1979, Field and Carrington, 2009). Antibody uptake occurs via hydrodynamic flow, and probably is independent of direct participation with cytoskeletal elements, elegantly explaining how GPI-anchored VSG-antibody complexes are efficiently and selectively targeted to the flagellar pocket (Engstler et al., 2007). The absence of AP2 is probably a specialization of trypanosomes expressing VSG, as AP2 is present in *T. cruzi* and *Leishmania* spp., but absent from all African trypanosomes. Remodeling of the endocytic system is also an intimate component of development of the parasite as it progresses between hosts (Natesan et al., 2007, 2010). Further, recent studies with an experimental trypanocide directed against *N*-myristoyltransferase that leads to endocytic defects may suggest that endocytosis is essential in vivo as well as in vitro (Allen et al., 2003; Frearson et al., 2010).

Extensive studies based on RNAi-mediated knockdown and immunolocalization have identified a cohort of proteins involved in endocytosis and recycling of VSG and other surface molecules, and together with ultrastructural studies have delineated endocytic pathways for VSG and invariant trans-membrane domain surface proteins (ISGs) (Allen et al., 2003, Hall et al., 2004a,b, 2005, Pal et al., 2003; Gabernet-Castello et al., 2009, García-Salcedo et al., 2004, Chung et al., 2008, Grünfelder et al., 2003). In brief, VSG is taken up via a clathrin/Rab5-dependent mechanism, sorted at an intracellular location that has yet to be fully defined, and recycled back to the flagellar pocket via Rab11-dependent recycling endosomes. ISGs (invariant Surface Glycoproteins) appear to be less efficiently recycled, and are extensively ubiquitylated and targeted to the lysosome for degradation (Chung et al., 2008, see Field and Carrington (2009) for review). Further, using ectopic expression of mutant isoforms of Rab4, Rab5A, Rab5B and Rab11 we have shown that Rab5A and Rab11 mediate the major pathways for uptake and recycling of VSG-bound immunoglobulin (Pal et al., 2003).

Despite these significant advances in determining the cell biology of the endocytic system, there is little information on the role of this pathway in vivo. This is unfortunate, as the ability to test the importance of uptake of VSG-antibody complexes and also the role of the pathway in sensitivity to other immune effectors, for example trypanosome lytic factor, remain to be determined in a physiological setting. This failure has been mainly due to the extreme phenotypes that arise following knockdown of many of the endocytic components, including the clathrin heavy chain, actin, epsinR and Rab5A and Rab11 (Allen et al., 2003; García-Salcedo et al., 2004; Gabernet-Castello et al., 2009, Hall et al., 2004a, 2004b, 2005). Each of these factors leads to significant loss of viability in vitro, confounding any attempt to separate the influence of the host environment from fundamental cellular functions. Using a clathrin heavy chain hemizygote with very moderate defects in endocytosis and several previously characterized cell lines expressing Rab mutant isoforms, we assessed the importance of endocytosis and recycling in a mouse model of infection.

## 2 Materials and methods

### 2.1 *Trypanosomes*, in vitro cultivation and mouse infections

Bloodstream form *Trypanosoma brucei brucei* MITat 1.2 (Lister 427) and procyclic form *T. b. brucei* MITat 1.2 were grown at 37 °C in HMI-9 or at 27 °C in SDM-79 respectively as previously described (Brun and Schönenberger, 1979; Hirumi and Hirumi, 1994). To estimate their ability to infect mice in vivo, wild type and manipulated BSF parasites were grown in ICR mice. Mice infected with wild type BSF were culled for humane reasons when parasitaemia was greater than 1 × 10^8^/ml. The level of parasitaemia was determined by tail bleed and counting parasites under a microscope over a period of two to seven days post-infection using a haemocytometer. All procedures involving animals and the housing of the animals were performed in accordance with the ethical guidelines of the University of Glasgow or Edinburgh.

### 2.2 Recombinant DNA manipulations

To overexpress clathrin heavy chain (CLH) in BSF and PCF cells, we PCR amplified the 5112 bp CLH ORF Tb10.70.0830 from wild type 427 genomic DNA using primers TbCLHFNdeI, GCCATATGATGGATAATCCACTAACCTCTGC, and TbCLHREcoRI, GCGAATTCTCAGTATGGCATCATGTTAGGG. Restriction sites are underlined. The PCR product was blunt cloned into pCR2.1-TOPO vector, and the CLH ORF released by digesting the pCR2.1-TOPO vector using NdeI and EcoRI and cloned into pXS5 and pXS2 to generate pXS5-CLH and pXS2-CLH respectively. Both pXS5-CLH and pXS2-CLH were fully sequence verified and linearized with XhoI or BstXI before electroporation with BSF or PCF parasites. Transfected BSF and PCFcells were grown in HMI-9 media containing 50 μg/ml neomycin, to isolate clathrin over-expressing lines. To generate a CLH single allele knockout construct ∼1 kb from the 5’ UTR of Tb10.70.0830 was PCR amplified using primers TbCLH5‘UTRF GCGGTACCTACACATAAGTGAAGGAGGG and TbCLH5‘UTRR GCCTGGAGCTTTGTTAGTGTCTGTTCC, and ∼1 kb from the 3’ UTR using primers TbCLH3’UTR-F GCACTAGTCACAGGGAAGGGAGATGGGA and TbCLH3’UTR-R GCGAGCTCGCAGCATTGGAAAGATGTGAG and blunt end cloned into pCR2.1-TOPO (Invitrogen). The 5’ UTR fragment was released from the pCR2.1-TOPO vector by digesting with KpnI and XhoI and cloned into pXS5:NEO or pXS2:NEO to generate pXS5-CLH5’UTR:NEO and pXS2-CLH5‘UTR:NEO, respectively. The 3’ UTR was released from the pCR2.1-TOPO vector by digesting with SpeI and SacI and cloned into pXS5-CLH5’UTR:NEO or pXS2-CLH5’UTR:NEO to generate pXS5-CLH5’3’UTR:NEO and pXS2-CLH5’3‘UTR:NEO, respectively. pXS5-CLH5’3’UTR:NEO and pXS2-CLH5’3’UTR:NEO were used to replace a single allele of CLH in the BSF and PCF genome, respectively. Both constructs were sequence verified and restriction digested with KpnI and SacI prior to electroporation with BSF or PCF parasites. Positive transformats were selected on HMI-9 media containing 50 μg/ml neomycin. All transgenic cell lines described here were cloned by limiting dilution prior to further analysis.

### 2.3 Quantitative real-time PCR

Total RNA from *T. brucei* BSF and PCF parasites were extracted using the Qiagen RNeasy mini kit. Synthesis of cDNA was performed in a 25 μl reaction volume with 2 μg RNA and oligo dT primers using the superscript II reverse transcriptase kit (Stratagene). Further, PCR amplification of a 125 bp fragment of clathrin (4286–4410 bp) was performed either under standard PCR conditions or in a reaction mixture containing cDNA and IQ-SyBr-green supermix using a mini-opticon instrument (BioRad) using the primers qRTCLHF ATACGTGCCCTCAAAACCTG and qRTCLHR GGATTCGAGGTATGGCAGAA.

### 2.4 Protein electrophoresis and western blotting

SDS lysates from 1 × 10^6^–1 × 10^7^ cells were separated on 12% SDS–polyacrylamide gels and wet-blotted onto PVDF membrane (Immobilon, Millipore, Bedford, MA), blocked with 5% milk in TBS-T (Tris-buffered saline, 0.5% Tween 20) for two hours at room temperature and probed with antibody to CLH at 1:1000, Rab5A at 1:1000, Rab11 at 1:2000 and BiP at 1:10,000 in 1% milk followed by HRP-conjugated goat anti-rabbit IgG (Sigma) or rabbit anti-mouse IgG (Sigma) at 1:10,000 dilution in 1% milk in TBS-T. Detection was by chemiluminescence and exposure to X-ray film (Kodak BioMax MR).

### 2.5 Southern blotting

Southern blotting was performed using 5 μg of genomic DNA isolated from BSF or PCF parasites in log phase (Medina-Acosta and Cross, 1993). Genomic DNA was digested with NaeI and NdeI, separated by electrophoresis and transferred to a nitrocellulose membrane and probed with specific probes for CLH and Neomycin. Hybridization and washing was done as described previously (Sambrook et al., 1989).

### 2.6 Cell cycle progression

Trypanosomes were harvested by centrifugation, washed with PBS and fixed with 4% PFA in ice-cold vPBS. Immunofluorescence was performed as described previously (Field et al., 2004). Specimens were analyzed on a Nikon Eclipse epifluorescence microscope equipped with a Hammamatsu CCD camera and data collected in Metamorph under non-saturating conditions (Molecular Devices). For determination of position in cell cycle cells were stained using DAPI, as described (Field et al., 2004); at least 200 cells were examined for each condition.

### 2.7 Transferin uptake assay

Mid-log phase BSF WT or BSF CLH-1KO cells from culture were harvested, washed and resuspended in serum-free HMI-9 containing 1% BSA at a concentration of ∼1 × 10^7^ cells/ml. Resuspended cells were incubated for 30 min at 37 °C and 125 μg/ml of Alexa-conjugated transferrin (Molecular Probes) was added. Aliquots were removed at 0, 5, 10, 15 and 20 min intervals after the addition of transferrin and placed immediately on ice. Cells were washed with vPBS, fixed with 1% formalin in PBS and transferrin accumulation was quantified using a CyAn ADP fluorescent activated cell sorter (DakoCytomation).

## 3 Results and discussion

### 3.1 Over-expression of CLH in BSF parasites is lethal

BSF parasites have a very high endocytic rate compared to the PCF parasites. We first attempted to increase endocytic rate still further by over-expressing CLH in both BSF and PCF parasites, by inserting the clathrin ORF into a ribosomal promoter region using the vector pXS5-CLH in BSF and into the inter-tubulin region using pXS2-CLH in PCF parasites (see Section 2). After electroporation of BSF with pXS5-CLH, we were unable to obtain neomycin resistant positive transformants after multiple attempts, despite efficient and successful transformation with control constructs. However we were able to obtain neomycin resistant positive transformants in PCFs after electroporation with pXS2-CLH (data not shown). Thus, these data suggest that over-expression of CLH in BSFs is not possible at this level, whereas augmentation of expression can be achieved in PCFs. This is consistent with previous work demonstrating increased clathrin protein expression in procyclic cells over-expressing Rab5A (Koumandou et al., 2008) and may suggest that the level of clathrin that is present in the BSF is the maximum that the cell can tolerate.

### 3.2 A single copy of CLH is sufficient for normal proliferation of BSF and PCF parasites

Having failed to augment expression of clathrin in BSFs, we turned to a single knockout approach, where we sought to generate hemizygote lines, in an attempt to partially down-regulate endocytosis. Single allele knockout mutants were generated by replacing the CLH ORF in both BSF and PCF parasites by homologous recombination of a construct containing the neomycin resistance gene flanked by ∼1 kb sequences from the 5’ and 3’ UTR of the CLH ORF. Positive transformants were selected by culturing transfected parasites with neomycin.

**Fig. 1:**
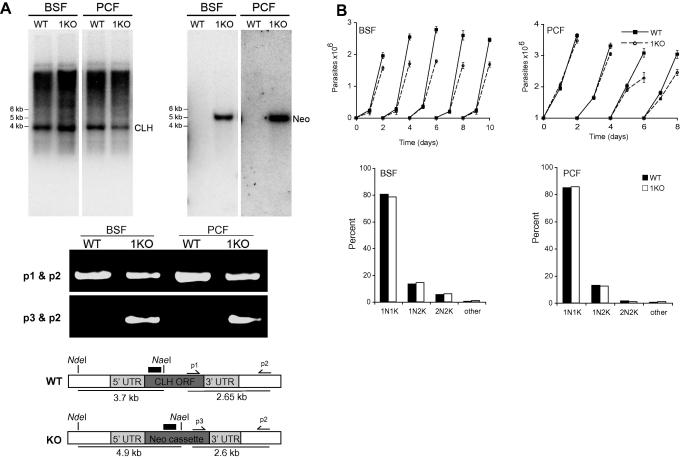
Generation and characterization of TbCLH hemizygotes. Panel A: Top inset; Southern blotting of genomic DNA confirming the knockout of one allele of CLH in both BSF and PCFs. 5 μg of genomic DNA from wild type parasites and single knockout mutants were restriction digested with NdeI and NaeI to generate a 3.7 kb fragment in the wild type allele (left panel) and a 4.9 kb fragment in the knockout allele (right panel). Hybridization with a specific probe for CLH and neomycin shows the presence of neomycin cassette at the correct molecular weight for authentic insertion (right panel). Positions of co-electrophoresed molecular weight markers are indicated at left of the panels. Middle inset; PCR for wild type allele using primers p1 and p2 resulted in a 2.65 kb product in both wild type and single knockout BSF and PCF parasites. However a 50% reduction in the amount of PCR products was observed in the BSF and PCF single knockout alleles confirming the loss of a single clathrin coding allele. PCR for knockout allele using primers p2 and p3 resulted in a 2.6 kb product only in the single knockout parasites. Bottom inset; schematic representation of the TbCLH locus before (427) and after (KO) integration of the neomycin knockout construct. Restriction sites and sizes of predicted fragments generated are shown underlined. Positions of the oligonucleotide probes used in the Southern blots are shown as thick black bars. Positions of primers used in PCRs and the size of predicted PCR products are represented. Panel B: TbCLH BSF (left panels) or PCF (right panels) hemizygotes exhibit minor defects in proliferation. Top: Growth curves of TbCLH hemizygotes (open symbols), compared to wild type parental lines (closed symbols). Cells were subcultured every 48 h. The experiment has been performed three times with the standard deviation between replicates shown. Bottom: TbCLH BSF or PCF hemizygotes exhibit no defects in cell cycle progression. Cells were stained with DAPI to visualize the kinetoplast and nucleus, and numbers of cells with the indicted organellar composition determined by fluorescence microscopy. Wild type parental cells are shown as closed symbols and the hemizygotes as open symbols.

To confirm deletion of a single copy of CLH in both BSF and PCF single allele knockouts, we performed Southern blotting. Digestion of wild type genomic DNA with enzymes NdeI and NaeI resulted in a ∼3.7 kb DNA fragment which includes a part of both the 5’ UTR and CLH ORF. However, in the single allele knockout mutants a 3.7 kb DNA fragment from the wild type allele and a 4.9 kb DNA fragment resulting from the knockout allele would be expected (Fig. 1A). Using specific probes targeting CLH and neomycin ORF regions in the excised DNA fragment, we were able to confirm the correct insertion of the knockout construct (Fig. 1A). In single allele knockout mutants we detected a decrease in hybridization signal intensity from the ∼3.7 kb DNA fragment representing CLH ORF when using the clathrin probe. On using the neomycin probe we were unable to detect any hybridization signal in the wild type cells, but were able to detect a ∼4.7 kb DNA fragment in the single allele knockout mutants confirming the deletion of CLH allele and the integration of neomycin cassette (Fig. 1A). In addition we performed PCR using genomic DNA as template from wild type and modified cells. Primer p1 (ATACGTGCCCTCAAAACCTG) corresponds to sequence within the clathrin coding region, primer p3 (CTATCAGGACATAGCGTTGG) to the neomycin coding region and primer p2 (ACGTTTCTCCCTTTCCCTCC) to ∼740 bp downstream to the clathrin 3’UTR region used for generation of the KO allele. Reactions using primers p1 and p2 amplified a 2.7 kbp product representing the wild type allele in both wild type and single knockout parasites (Fig. 1A). PCR with primers p2 and p3 resulted in a 2.6 kbp product only in the single knockout parasites representing the knockout allele, confirming the single allele knockout (Fig. 1A). Deletion of a single CLH allele from BSF and PCF parasites generated only a minor defect in the proliferation of these cells in culture (Fig. 1B). However there was no significant defect to cell cycle progression (Fig. 1B) suggesting that removal of one CLH allele did not perturb mitosis or cytokinesis.

### 3.3 Endocytosis is down-regulated in BSF single allele knockout mutant but their ability to infect mouse is unaltered

**Fig. 2:**
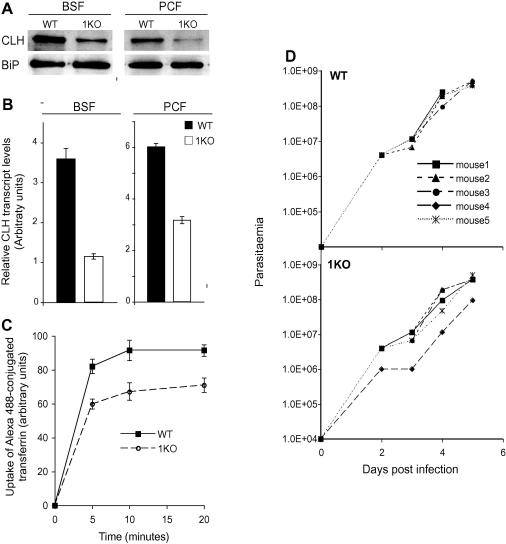
Expression of clathrin heavy chain mRNA and protein and in vivo phenotype of TbCLH hemizygotes. Panel A: Western blot using 1 × 10^6^ cell equivalents demonstrating the down-regulation of TbCLH protein in both BSF and PCF hemizygotes. The ER chaperone BiP is expressed equally in both wild type and hemizygote parasites. Representative experiment of three. Panel B: qRT-PCR analysis of TbCLH mRNA levels, using total RNA obtained from wild type and hemizygote parasites, indicate a down-regulation of CLH in hemizygotes. The experiment has been preformed twice with the standard error indicated. Wild type data are black bars, hemizygote data open bars. Panel C: Uptake of Alexa488-conjugated transferrin in both wild type and TbCLH hemizygote parasites. Wild type data in black symbols, hemizygote data in open symbols. Accumulation reached a maximum by 10 min, but there is a significant reduction in the amounts of Alexa488-conjugated transferrin endocytosed by the CLH-1KO parasites, suggesting down-regulation in endocytosis. Representative data from three experiments. Panel D: A single allele of TbCLH is sufficient to maintain normal infection rates in mouse. 1 × 10^4^ wild type or TbCLH hemizygote BSF parasites were used to infect five different mice in each cohort. Parasitaemia was determined by tail bleed from two to five days post-infection and data from individual mice are shown. There was no significant difference in the ability of the TbCLH hemizygotes to infect mice compared to the wild type parasites.

To both confirm and characterize the CHC single allele knockout mutants we measured the levels of CLH expression. By western blotting we found the amounts of CLH in both BSF and PCF hemizygotes reduced to ∼50% compared to the wild type while there was no change in the amounts of BiP, an ER marker (Fig. 2A). We also performed quantitative RT-PCR with RNA extracted from wild type and hemizygote parasites and again detected a significant decrease in the levels of CLH transcripts (Fig. 2B). Together these data indicate that we have indeed generated a single knockout, that this resulted in decreased clathrin protein, but that this was compatible with viability, i.e. clathrin is haplo-sufficient. Importantly, we did not detect an increase in the number of cells with enlarged flagellar pockets (BigEye cells, Allen et al., 2003) in the BSF hemizygote, a hallmark of severely depressed clathrin expression and resulting from imbalance of bulk membrane flux though the flagellar pocket (Allen et al., 2003). However, we previously noted that the BigEye phenotype was only detected in BSF cells where the majority of clathrin protein had been lost (Allen et al., 2003), suggesting that a threshold has to be reached before the onset of morphological defects; presumably the single allele in the hemizygotes is sufficient to avoid this.

To investigate if decreased clathrin expression altered endocytic activity we analyzed the ability of BSF CLH hemizygote cells to endocytose Alexa488-conjugated transferrin and quantified uptake by fluorescence-activated cell sorting (FACS). In both wild type and CLH hemizygotes transferrin accumulation reached a maximum by ten minutes (Fig. 2C). However there was a significant reduction in the total amount of transferrin accumulated, with ∼25% less in the hemizygote cells compared to wild type (Fig. 2C). We interpret the decrease in steady state levels of transferrin to slower uptake rates in the presence of an unaltered degradation rate (Pal et al., 2003; Mackey et al. 2004; O’Brien et al. 2008). Therefore, despite the absence of a clear impact on bulk membrane flow a small but significant effect in receptor-mediated endocytosis is found in the hemizygote cells; the uncoupling of specific protein from bulk membrane endocytosis is similar to that previously observed for epsinR, where decreased endocytosis of several proteins was found but the BigEye morphology was not observed (Gabernet-Castello et al. 2009).

Next we investigated if the reduced clathrin expression in the hemizygote cells was sufficient for infection or proliferation in the mammalian host by inoculating mice with low numbers of parasites (1 × 10^4^ cells). Wild type BSF parasites produced a parasitaemia of ∼1 × 10^9^ parasites ml^−1^ five days post-infection (Fig. 2D). Similarly, the hemizygote cells were able to cause parasitaemia without any significant difference to the wild type for four of five mice, and a minor impact on infection in only one animal (Fig. 2D). Therefore while the hemizygote cells have reduced levels of clathrin, which is reflected in decreased transferrin accumulation, this level is still sufficient for robust growth in mice.

### 3.4 Rab11 is important in infection of the mammalian host

In bloodstream trypanosomes several small GTPases (Rabs) mediate various endocytic pathways. Rab4 regulates lysosomal delivery, while Rab5A and Rab11 are involved in early endocytosis and recycling respectively (Pal et al., 2003; Hall et al., 2004a, 2004b, 2005). Over-expression of Rab5A and Rab11 mutants in bloodstream cells leads to defects in trafficking of transferrin and anti-VSG antibodies, while manipulation of Rab4 has little impact (Pal et al., 2003).

**Fig. 3:**
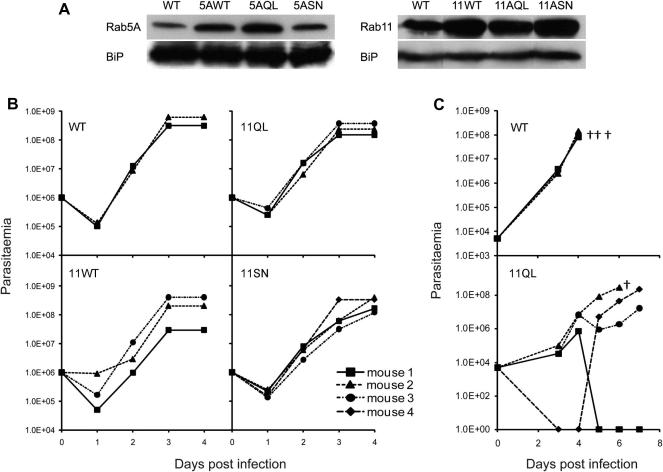
Over-expression of the TbRab11QL variant reduces the ability of BSF parasites to infect mice. Panel A: Characterization of BSF parasites over-expressing Rab5A and Rab11. Western blots using 1 × 10^7^ cells demonstrating the over-expression of WT, QL and SN isoforms of both TbRab5A and TbRab11 in transgenic parasites. TbBiP, an ER protein, remains unchanged across the parasite lines. Panel B: Over-expression of TbRab11 isoform in BSF parasites reduces the ability of BSF parasites to infect mice. Left panels; Inoculation of 1 × 10^6^ BSF parasites over-expressing TbRab11 WT, QL and SN isoforms in mice demonstrates normal levels of parasitaemia. Panel C; Inoculation of 5 × 10^3^ BSF parasites over-expressing the TbRab11QL isoform in mice attenuates infectivity. Other isoforms did not exhibit altered levels of parasitaemia and no attenuation of infectivity/survival in mice was obtained with any of the Rab5 isoform cell lines. The experiment was performed three times with cohorts of four or five mice in each instance, and a representative graph is presented. † mice were culled if parasitaemia was greater than 1 × 10^8^ cells/ml.

To investigate involvement of Rab4, Rab5A and Rab11 in mammalian infectivity we over expressed both the wild type (WT), dominant active mutant (QL) and inactive mutant (SN) forms of all the three Rab proteins in trypanosomes and then infected mice. We used Rab4 lines generated previously (Pal et al., 2003) but recreated the Rab5 and Rab11 lines by transfecting trypanosomes with the respective DNA constructs from that earlier study (Pal et al. 2003); we validated these new lines by Western blotting (Fig. 3A) and in all cases over-expression similar to our earlier work was obtained.

When we infected mice with high numbers of the transgenic cells, i.e. 1 × 10^6^ cells per mouse, there was no significant change to parasitaemia between wild type and cells expressing Rab mutant isoforms (data not shown and Fig. 3B). However, when infected with lower numbers, i.e. 5 × 10^3^ cells per animal, wild type trypanosomes proliferated rapidly, and mice were culled for humane reasons after 4 days when parasitaemia reached >1 × 108 cells/ml (Fig. 3C, top). Except Rab11QL-expressing cells, similar growth kinetics were observed for all trangenic lines investigated (data not shown and Fig. 3C, lower). This Rab11QL-expressing cell line exhibited less robust proliferation and four of five infected mice were still alive after six days. Two mice survived a further day, indicating that over-expression of Rab11QL in BSF trypanosomes reduced the ability of the parasites to proliferate in the mammalian host.

It is unlikely that the effect seen here is due to turnover of immunoglobulin, as the period of infection is too short for a robust immune response to have been mounted in the mice. However, it is known that the Rab11 compartment contains several highly active metacaspases (Helms et al., 2006), which may suggest that their function, or that of some other Rab11 compartment resident, is disrupted in these cells, albeit without a clear defect in in vitro culture. This is consistent with the observation that null mutants can be generated through deletion of three of five metacaspase genes (MCA2, MCA3 and MCA5), but trypanosomes are not viable if RNAi is performed against all three genes, suggesting that the parasite can adapt if the insult is not too severe (Helms et al., 2006).

## 4 Conclusions

Endocytosis in African trypanosomes is essential, and demonstrated via numerous genetic approaches. The pathway is implicated in immune evasion by virtue of providing a mechanism for removal of surface bound immunoglobulins recognizing VSG. The exclusive focus of endocytosis/exocytosis at the flagellar pocket, together with the extreme membrane trafficking flux in the mammalian infective stage most likely represents an excellent drug target. We sought to separate the essential nature of expression of clathrin and Rab proteins mediating endocytosis from host-parasite interactions by invoking mild alterations in expression or function. Unexpectedly, clathrin expression and Rab5 activity could be decreased without compromising infectivity, but the recycling pathway mediated by Rab11 was important for survival of parasites within the host. The absence of an influence for the uptake arm of the endocytic system suggests that in the bloodstream form endocytosis functions at such a high level that, in the absence of an immune response, there is more than sufficient activity. It will be of interest to examine the effects of these mutations in models where more chronic infections can be established or in knockout mice where specific components of the adaptive immune system have been deleted, and hence to determine if cells bearing altered trafficking pathways are able to survive in the presence of antibodies recognizing surface molecules.
